# Early Sensitivity to Morphology in Beginning Readers of Arabic

**DOI:** 10.3389/fpsyg.2020.552315

**Published:** 2020-09-23

**Authors:** Carole El Akiki, Alain Content

**Affiliations:** ^1^Laboratoire Cognition Langage et Développement, Center of Research in Cognition & Neurosciences, ULB Neuroscience Institute, Université libre de Bruxelles (ULB), Brussels, Belgium; ^2^Speech Therapy Department, Faculty of Public Health II, Lebanese University, Fanar, Lebanon

**Keywords:** Arabic language, reading acquisition, morphology, morphological awareness, root/pattern structure

## Abstract

The current study investigated the influence of morphological structure on the earliest stages of Arabic reading acquisition. More specifically, we aimed at examining the role of root and pattern units in beginners from Grade 1 to 3. A first set of reading tasks evaluated the presence of a morphology facilitation effect in word and pseudoword reading by manipulating independently the frequency of roots and patterns. Additional tasks aimed at examining the contribution of morphological awareness to reading performance. The results suggest that reading ability is early influenced by the awareness of morphological composition. Children read faster and more accurately pseudowords composed of frequent morphemes. Furthermore, regression analyses revealed, for every reading measure, a significant contribution of one morphological test in addition to grapheme knowledge. Results are discussed taking into account the differences obtained depending on lexicality and morpheme type (root or pattern).

## Introduction

Reading acquisition entails the establishment of connections between orthography, phonology and meaning. Until recent years, most models have assumed that the major component of early stages of reading acquisition consists in the development of a phonological recoding system based on grapheme–phoneme correspondences (GPCs) (Frith, 1985; Ehri and McCormick, 1998; Seymour et al., 2003; Sprenger-Charolles et al., 2003; Ehri, 2005; Ziegler and Goswami, 2005). Associations between larger orthographic and phonological units, such as morphemes, were deemed to appear later in the development. However, recent evidence has accumulated to indicate the contribution of morphology already in beginning readers (Burani et al., 2002; Quémart et al., 2011; Beyersmann et al., 2015, 2019; Casalis et al., 2015; Hasenäcker et al., 2017; Dawson et al., 2018).

The present study aimed at examining the role of morphology in the first years of reading acquisition in Arabic. Studies on different languages prove essential to ensure the generality of theories (Share, 2008), and a universal model of reading and reading acquisition would require a detailed account of how the orthographic and linguistic features of different languages determine the mechanisms of word recognition and their learning (Frost, 2006, 2012). In this perspective, the Arabic language and writing system constitutes an interesting case to explore. Arabic belongs to the group of Semitic languages, such as Aramaic, Hebrew and Syriac. Its writing system, called Abjad, contains 28 consonant graphemes and three vowel graphemes representing the long vowels ا/ā/, و/ū/, and ي/ī/. As the script is cursive, graphemes are ligated, and they may receive different graphic forms, as a function of position and orthographic context. In addition, the three short vowels are represented by diacritical marks appearing above the line, َ–/a/, ُ–/u/, or under the line, ِ–/i/.

Several features of the Arabic language and writing system are especially relevant to the issue of the role of morphology in reading acquisition. First, Arabic texts can be written in two different forms, with or without the diacritics representing the short vowels. Short vowels are usually omitted, except for didactic purposes in children’s books. When they are absent, readers need to resort to lexical morpho-orthographic knowledge as well as syntactic and semantic information to retrieve the correct pronunciation (Mahfoudhi et al., 2011; Saiegh-Haddad and Henkin-Roitfarb, 2014). For instance, the unvowelled form كتب could correspond either to the word/kataba/, كَتَبَ, *he wrote* or/kutub/, كُتُب, *books.* In contrast, vowelled texts with the short vowel marks, as used during the first primary school years, are phonologically transparent from the point of view of reading.

Secondly, GPCs in Arabic are generally consistent, except for a few peculiarities (Saiegh-Haddad and Henkin-Roitfarb, 2014; Asadi et al., 2017a). Thus, one might assume that consistency favors the development of a simple GPC procedure. However, rapid early acquisition of the GPC system might facilitate access to larger orthographic units (Ziegler and Goswami, 2005), which would later become critical to process unvowelled writing.

Thirdly, like other Semitic languages, Arabic features a very rich non-concatenative morphology. Word forms combine a consonantal root and a word pattern. The root is most often triliteral but can also consist of two or four consonants, and it indicates the semantic field of the word. Word patterns are phonological templates including the vowels interspersed among the root elements. Consequently, morphemes are discontinuous and there is no linear correspondence between the morphological structure and either the phonological or the orthographic segmentation (Holes, 2004; Saiegh-Haddad, 2004; Ryding, 2005). Neither the consonantal skeleton nor the word pattern are ever encountered in isolation, and neither have an independent lexical status or can be pronounced separately because roots are always inserted within a given word pattern (Blachère and Gaudefroy-Demombynes, 1975). In addition to the vowels, word patterns may entail gemination of some of the root consonants, and they may be augmented by affixes. For instance, the word/kātib/, كاتب, *writer*, is formed by the combination of the root {ktb}^1^ and the word pattern {CāCiC}^2^ whereas/maktūb/, مكتوب, *written*, has the same root and the pattern {maCCūC} with the prefix/ma/.

Word formation principles include both inflectional and derivational/lexical processes. Derivational processes consist in the combination of a root with different patterns, to produce lexical items varying in their meaning or grammatical class while sharing the semantic field characteristic of the root. For instance, the words ملعب, /malʕab/, *playroom*, and اعب لِ, /lāʕeb/, *player*, which share the {lʕb} root referring to the domain of ‘play,’ designate respectively the location and the agent. Over and above the derivational mechanisms that give rise to word forms, inflectional affixes are added to base forms to indicate tense (/darasa/, دَرَسَ, *he studied* vs./jadrusu/, يَدرُسُ, *he studies*), gender (ملِكَة - مَلِك, /malik/-/malikat/, *king* - *queen*) or number (رَسّامون - رَسّام, /rassām/-/rassāmūn/, *painter - painters*) (Ryding, 2005).

In sum, one essential feature of Arabic is that the lexicon is structured in terms of around 5,000 consonantal roots (Ryding, 2005; Boudelaa and Marslen-Wilson, 2010), and a much smaller number of patterns, from which word forms are derived. This structure is made more salient by the writing system which highlights consonants. The rich root/pattern morphological structure of Arabic might play a role in speech and reading processes, which prompted researchers to investigate the organization of the mental lexicon in Arabic-speaking readers. There is now clear evidence for a contribution of morphology to reading in expert Arabic readers (Boudelaa and Marslen-Wilson, 2001, 2005, 2011; Boudelaa, 2014).

As regards reading acquisition, the richness of the morphology and the saliency and quasi-systematic occurrence of the root/pattern structure might induce sensitivity to word composition and encourage beginners to exploit it. In addition, the morphological structure may help readers recover the phonological information when diacritics are absent in unvowelled Arabic, as mentioned above. By contrast, the discontinuous nature of morphological elements and the ligated script might make it harder for young children to isolate and manipulate separate morphemes (Bar-on et al., 2018). In a review of the literature, Saiegh-Haddad (2018) proposed a Model of Arabic Word Reading (MAWRID) in which she draw attention to the influence of morphological structure in the development of word reading in Arabic.

Two empirical approaches have been exploited to investigate the role of morphology in literacy acquisition. The first stems from studies in which the morphological composition of the items to be read is manipulated. Few studies have explored this avenue in Arabic until recently.

Bar-on et al. (2018) investigated the contribution of the phonological information provided by vowel diacritics and the morphological information provided by morpho-orthographic root-and-pattern structure to oral reading of pseudowords. With unvowelled pseudowords combining pseudoroots with real patterns, even the youngest participants (2nd graders) produced about 75% correct pattern completions, suggesting that they exploited their knowledge of possible patterns. These results are in line with those of Bar-On and Ravid (2011) in Hebrew and thus suggest that morphology contributes to oral reading, as early as second grade. More specifically, this study highlights the important role of word patterns in word identification. However, as the authors note, the rate of valid pattern completions may be overestimated, due to a high proportion of homography (most unvowelled pseudowords being compatible with several pattern completions) and to the limited number of potential completions, as there are only three short vowels in Arabic. A more direct approach, which will be used in the present study, consists in comparing the performance on pseudowords comprising either frequent patterns or rare/non-existent ones. Saiegh-Haddad and Schiff (2016) and Schiff and Saiegh-Haddad (2017) found that second graders benefit from the presence of vowel diacritics, which presumably facilitate phonological decoding, whereas older participants more readily exploit their lexico-orthographic and morpho-orthographic competences to complete the missing information in unvowelled stimuli. Moreover, Taha and Saiegh-Haddad (2017) found that children take advantage of morphological structure to spell words and pseudowords. Additional evidence comes from priming studies. Shalhoub-Awwad and Leikin (2016) used the cross-modal priming paradigm in a lexical decision task to examine the effects of the root in second and fifth graders. Root primes sped up the identification of the target word and improved accuracy for both groups of participants. Results obtained in Hebrew similarly support the view that, very early in development, the visual mental lexicon is organized on morphemic basis (Schiff et al., 2012). Overall, to our knowledge, no available study examined systematically the developmental course of the sensitivity to root and to pattern frequency in oral reading.

The second approach consists in assessing the relationship between morphological awareness (MA) and reading development. MA is defined as the ability to reflect on and manipulate the constituent morphemes of words (Carlisle, 1995). With Indo-European languages, correlational studies have provided evidence that MA is linked to literacy development (e.g., Bowers et al., 2010; Marec-Breton et al., 2010). In Arabic reading acquisition, MA seems to play some role, although phonological skills constitute the key component (Abu-Rabia et al., 2003; Saiegh-Haddad, 2003, 2005; Elbeheri and Everatt, 2007; Taibah and Haynes, 2011; Taha, 2013; Saiegh-Haddad and Taha, 2017). Empirically, MA was found to contribute to word reading (Abu-Rabia et al., 2003; Abu-Rabia, 2007; Saiegh-Haddad and Geva, 2008; Boukadida et al., 2009; Abu-Ahmad et al., 2014; Tibi and Kirby, 2017, 2019), pseudoword reading (Tibi and Kirby, 2017, 2019) and reading comprehension (Abu-Rabia et al., 2003; Abu-Rabia, 2007; Mahfoudhi et al., 2010; Asadi et al., 2017c; Tibi and Kirby, 2017, 2019). However, some studies failed to show the association (Abu-Rabia and Abu-Rahmoun, 2012; Asadi et al., 2017b; Layes et al., 2017). As argued by Marec-Breton et al. (2010), the strength of the link between MA and reading may differ according to the type of knowledge considered and this could explain the inconsistencies between studies, which vary in the nature of the morphological tasks (analogy, judgment, production, etc.), the type of morphology (derivational and/or inflectional), the morphemes that are targeted (root and/or pattern) as well as the measures of reading ability (reading words, pseudowords and/or comprehension). It is worth noting that most studies in Arabic used words to assess MA, making it difficult to isolate knowledge of morphology from lexical knowledge, especially when vocabulary was not assessed. Moreover, not all studies controlled for the influence of relevant predictors such as IQ, vocabulary, letter knowledge, or phonological awareness, with only a few exceptions (e.g., Asadi et al., 2017b, c; Tibi and Kirby, 2019). In the present study, MA was assessed with pseudowords to avoid vocabulary confounds, and we controlled for the major reading predictors.

The goal of our study was to investigate in more detail the influence of morphological structure on the earliest stages of reading acquisition. More specifically, we aimed at examining the role of root and pattern units in beginners from Grade 1 to 3. We adopted two complementary approaches. The first was experimental. We manipulated independently the frequency of roots and patterns to evaluate their impact on oral reading. If morphological analysis is implicated in word recognition, then frequency of the root and the pattern should facilitate word and pseudoword reading. Although the manipulation of the frequency of morphemic components has been one standard approach in the study of adult reading processes (Ford et al., 2010), this is, to our knowledge, the first attempt at exploiting such a technique for both root and pattern morphemes with children in Arabic. Roots and patterns could have different roles in reading acquisition. While there is growing evidence that root identification may facilitate reading both in terms of speed and accuracy, patterns are critically important to retrieve the phonological information missing in unvowelled orthography (Saiegh-Haddad and Schiff, 2016; Schiff and Saiegh-Haddad, 2017; Bar-on et al., 2018).

The second approach was correlational and sought to assess the links between MA and reading performance. We aimed at examining whether tests of MA predict word, pseudoword decoding and word comprehension. To that end, several tests were designed to assess general cognitive ability (non-verbal IQ, working memory span, vocabulary) and known predictors of reading acquisition, namely phoneme discrimination, phonological awareness (phoneme deletion), rapid naming, and knowledge of letter sounds. Evaluation of children’s awareness of morphology involved two induction tests that required to apply known morphological processes to produce new forms, as in Berko’s (1958) paradigm. Children were required to derive a pseudoword from the one provided by the experimenter, according to a manipulation rule conveyed through examples. We assume that such tasks require awareness of morpho-phonological and morpho-semantic relationships in order to extract the change rule, and also morpho-phonological manipulation ability in order to generate the target response from the stimuli. While correlational evidence of a contribution of MA to reading acquisition in Arabic is already available, we reasoned that induction tasks with pseudowords might constitute an alternative and purer way to assess MA, the ability to reflect upon and manipulate morphological constituents, and we expected that the convergence of the two approaches would provide stronger evidence to the role of morphology in reading acquisition.

## Materials and Methods

### Participants

A total of 139 pupils from three private primary schools in Lebanon participated in this study, 22 from Grade 1, 59 from Grade 2, and 58 from Grade 3 (see details in Table 1).^3^ Based on parents’ occupation and school fees, families were from medium socioeconomic status. Participants were recruited on a voluntary basis with parental permission. All children in the classes participated, except those who were or had been undergoing speech or psychomotor therapy. All were children following the regular curriculum in bilingual schools without reported history of language disorders or learning disabilities. They had spoken Arabic as their first language and learned modern standard Arabic (MSA) and written French in school, starting in kindergarten. Some had been exposed to French as a second language in the nursery or in preschool, others at home.

**TABLE 1:** Descriptive characteristics of the participant sample (SD in brackets).

	Grade 1	Grade 2	Grade 3
N participants	22	59	58
N boys/girls	8/14	29/30	26/32
Age (months)	79.6 (4.0)	90.4 (4.6)	102.4 (5.3)
Raven – percent correct	60.5 (11.2)	67.7 (13.1)	70.4 (11.8)
Vocabulary – percent correct	77.9 (8.06)	83.4 (9.66)	90.2 (4.94)
Forward span (max 15)	7.64 (1.76)	7.69 (1.26)	8.29 (1.88)
Backward span (max 15)	4.23 (1.38)	4.03 (1.17)	4.86 (1.67)

The study was approved by the Ethics Committee of the Faculty of Psychological Sciences of the Université libre de Bruxelles before the start of data acquisition.

### Tasks

In addition to the word recognition and MA tests, all children received the Raven Matrices test, a receptive vocabulary test adapted from the EVIP (Dunn et al., 1993), forward and backward digit spans, and tests of letter knowledge and phonological abilities.

#### Word Recognition

##### Word and pseudoword reading

Four lists of bisyllabic pseudowords were created, each composed of ten items. All stimuli were matched across the four lists in number of letters, number of phonemes as well as syllabic structure (see Supplementary Material). Pseudowords were constructed by combining either frequent roots (R+, e.g., {ktb}, “write”) or rare/non-existent roots (R−, e.g., {dbs}, “sweeten”) with either frequent nominal patterns (P+, e.g., {CaCāC}) or rare/non-existent ones (P−, e.g., {CaCCīC}), resulting in four lists, R+P+ (e.g., طَليب/ṫalīb/), R+P− (e.g., هيرَب/hīrab/), R−P− (e.g., لوماز/lūmāz/) and R−P+ (e.g., باسِك/bāsik/). Root and pattern frequencies were taken from the Aralex lexical database (Boudelaa and Marslen-Wilson, 2010), which provides constituents’ frequency estimates based on a sufficiently large corpus of texts. Due to the other matching constraints, pseudoroots and pseudopatterns had to be included. The same design was adopted for words. However, the R+P− condition could not be created as it was impossible to find appropriate items to match with the three other conditions. Words were selected to be of medium to high frequency, as well as relatively familiar to young children. Because the Aralex frequency counts are based on adult reading materials and may not be reliable for studies with children, the initial selection of words relied on a database of word frequency compiled by the first author, based on the most used Grade 2 and 3 schoolbooks in Lebanon. As an additional validation, twenty-five pupils from Grade 2 and 3, not included in the main study, were asked to estimate word familiarity on a scale from 1 (“words that I see very rarely”) to 4 (“words that I see frequently”). The mean familiarity ratings for each of the three lists (R+P+, R−P+, and R−P−) were 2.99, 2.94, and 2.95 respectively (see details in Supplementary Material and stimuli in Annex 1).

All stimuli were presented in vowelled form. Children were required to read aloud each of the seven lists as fast and accurately as possible. Oral responses were encoded by the experimenter and recorded for later checking, and the total time spent on each list was noted. Reliability across the seven word and pseudoword lists was high, α = 0.91 for correct responses and 0.94 for efficiency scores.

##### Written word comprehension

In order to evaluate access to meaning, a speeded semantic categorization test was devised. Three lists of 15 familiar words each were presented, including respectively seven clothing item names, seven fruits and vegetable names and six animal names (see Annex 2). Children were asked to read each list silently and underline the words belonging to the instructed category, and the total time to process each of the three lists was recorded. Reliability across the three word lists was 0.55 for correct responses and 0.92 for efficiency scores.

#### Morphological Awareness

##### Morpho-semantic induction

This test was inspired by Berko’s (1958) study of morphological knowledge in young children. It required to change the morphological pattern of pseudowords. Six patterns were chosen, three involving inflections and three involving derivations. For each of the six change paradigms, three examples were provided with words, to inform about the semantics of the transformation (e.g., derivational paradigm, “agent,”/huwa rasama - ʔinnahu *rassām*/“he paints, he is *a painter*”; inflectional paradigm, “plural,”/huwa jadrusu - hum *jadrusūn*/, “he studies, they *study*”). Then eight trials with pseudowords were presented (for instance, derivational paradigm, “agent,”/huwa falaba - ʔinnahu *fallāb*/, inflectional paradigm, “plural,”/huwa jaʕsibu - hum *jaʕsibūn*/).

The inflectional patterns selected appear very early in school textbooks and were thus familiar to children. The derivational patterns were also quite frequent according to Aralex (see Annex 3). In addition, we manipulated root frequency, which alternated within each paradigm. Half the trials involved a frequent root and half a rare one. The child had first to repeat the stimulus and then produce the transformed pseudoword. Feedback was systematically provided at each trial, whether the answer was correct or not. Children were allowed an interval of 10 s to respond. In case of abstention, the experimenter produced the correct response. To familiarize children with the task, four trials based on a different paradigm were provided before the beginning of the actual test. Reliability across the four conditions was 0.68.

##### Morpho-phonological induction

We constructed a second induction situation in which children were to perform complex operations similar to those existing in morphological paradigms of the Arabic language. To isolate the phonological component of the morphological manipulations, the examples were presented with a neutral instruction so that no semantic context was provided (e.g., if I say/rahasa/, you have to say/rāhis/*;* if I say/daraba/, you have to say ….). Both the stimuli and the responses were pseudowords. To ensure that the task taps into morphological knowledge, the frequency of the roots and the patterns were manipulated. If children explicitly use their morphological knowledge to extract the transformation rule and perform the manipulation, performance should vary with constituents’ frequency. Six paradigms were devised, three of which used frequent word patterns and the three others used word patterns that do not exist in Arabic. Frequent and non-existent paradigms were chosen to be as similar as possible in terms of stimulus and response length and complexity as well as number of phonological manipulations. For each paradigm, one example was given by the examiner, followed by eight trials, half involving a frequent root and half a rare one. The child had first to repeat the stimulus and then produce the transformed pseudoword. Feedback was systematically provided at each trial, whether the answer was correct or not. Children were allowed an interval of 10 s to respond. In case of abstention, the experimenter gave the correct response. To familiarize children with the task, a first series of four trials based on a different paradigm was provided before the actual test (see Annex 4). Reliability for the correct response rates across the four conditions was 0.91.

#### Control Variables

##### Letter naming

Thirty letter shapes (see Annex 5) were presented by series of five on cards. The letters were presented in ligated form, as in the initial, internal, or final position. All letters were included. Children were requested to name them as quickly and accurately as possible. Response accuracy and total time per card were recorded. Reliability (Cronbach alpha) across the five lists was high, α = 0.76 for correct responses and 0.90 for efficiency scores.

##### Phoneme discrimination

This test comprised 62 pairs of CVC nonsense syllables. Half the pairs were identical, and the other half differed by one phoneme. All syllables were attested in Arabic. Different types of contrasts were tested (see Annex 6). Syllables were recorded by a native speaker of Arabic and were presented with a 250 ms interval between the two items in a pair, and a 3-s response interval. Six examples were given prior to the test. Children had to judge whether the elements of each pair were identical or different. Split-half reliability was 0.51 for same pairs and 0.77 for different pairs.

##### Phoneme deletion

Three lists of ten pseudowords were created. The first two involved initial consonant deletion and were composed of CVC nonsense syllables with long or short vowels. The third used CVCC syllables of which the pre-final consonant had to be deleted (see Annex 7). All stimuli and responses were attested in Arabic. Each list was preceded by four examples with corrective feedback. Children had to repeat the stimulus and then produce the result of the deletion. No corrective feedback was provided during the test trials. Reliability across all 30 items was 0.86 but lower across the three lists (α = 0.66).

##### Rapid naming

This test was made of four boards, each with 24 pictures organized in four lines of six elements. Two boards comprised three images repeated eight times, and the other comprised 24 different images chosen from an online database for children. Participants’ productions for each board were timed and recorded. The first of the two boards in each condition was considered a practice trial (see Annex 8). Cronbach alpha for efficiency scores across the two lists was 0.61.

### Procedure

For all newly designed tasks, pilot testing was conducted with 30 second and third graders (not included in the main study) to ensure clarity of instructions, identify problematic items, and assess test duration. The pilot data are not included in the present report. Testing was carried out by the first author and three speech therapy Master students. Testing was performed during school hours over three individual sessions of 30 to 40 min each, in a fixed order, with a maximum interval of 1 week between the three sessions (December to February for Grade 2 and Grade 3, February for first graders). During the first session, the children took the Raven Progressive Matrices (Raven et al., 1998), receptive vocabulary, and the rapid naming test. The second session was devoted to word reading, letter naming, morpho-semantic induction and auditory discrimination. The third session consisted of pseudoword reading, phoneme deletion, morpho-phonological induction, memory span, and written word comprehension.

## Results

For timed tests the main analyses were based on *efficiency scores*, computed as the number of correct responses per second. Efficiency scores (or their inverse, mean response time/proportion correct) have been proposed and used for a long time to provide a performance measure combining speed and accuracy information (Townsend and Ashby, 1978, see Bruyer and Brysbaert, 2011 and Vandierendonck, 2017, for reviews and discussions). In addition to the advantage of offering a unique index of performance, efficiency scores seemed appropriate in the present case as data collection made it possible to gather only one estimate of response speed per condition, the total time spent for a given stimulus list. As recommended (Bruyer and Brysbaert, 2011; Vandierendonck, 2017), additional analyses on the rate of correct responses were also performed. For the other tests, the dependent measure was the percentage correct. Statistical analyses of control variables can be found in Supplementary Material. All statistical analyses were performed with JASP (JASP Team, 2019) and lme4 package (version 1.1-23, Bates et al., 2015) with R (version 4.0.0, R Core Team, 2020). Preliminary analyses included gender, which proved to be non-significant in all tests. Hence gender was not considered in the reported analyses.

### Word Recognition

#### Word and Pseudoword Reading

Analyses of variance were carried out on efficiency scores, with grade as a between-subject factor and lexicality (words vs. pseudowords), root frequency (R+ vs. R−), and pattern frequency (P+ vs. P−) as within-subject factors. Additional analyses on correct responses using mixed models were also performed, with participants and items as crossed random effects. Accuracy, coded as 0 or 1, was the dependent variable, and a generalized linear model was applied with the binomial logistic link function. Grade was coded with two dummy variables identifying Grade 2 and Grade 3 relative to Grade 1, and the other dichotomous predictors were contrast-coded.

**FIGURE 1:**
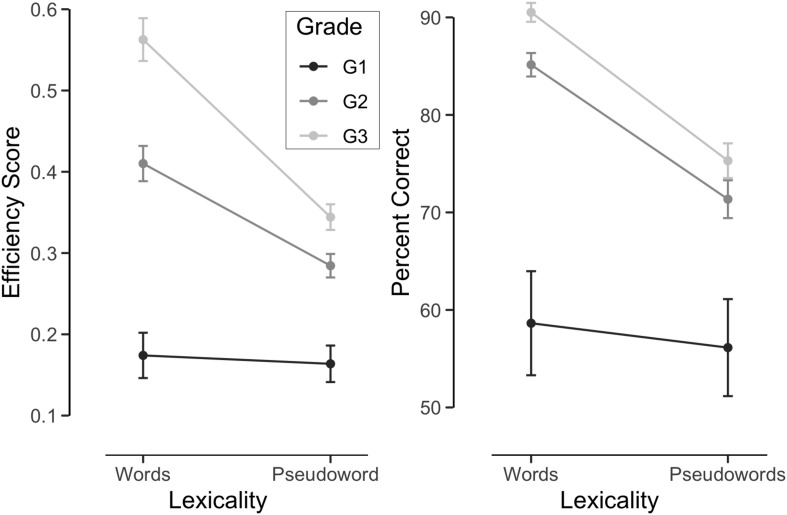
Reading efficiency and accuracy as a function of lexicality and grade.

A first ANOVA examined reading efficiency according to lexicality and grade (see Figure 1). Efficiency increased with grade, *F*(2,136) = 36.25, *p* < 0.001, $\eta_{\text{p}}^{2}$ = 0.35 and the lexicality effect was significant, *F*(1,136) = 98.55, *p* < 0.001, $\eta_{\text{p}}^{2}$ = 0.42. In addition, the presence of a lexicality by grade interaction, *F*(2,136) = 22.90, *p* < 0.001, $\eta_{\text{p}}^{2}$ = 0.25 showed that the lexicality effect varied with grade level. No significant lexicality effect was present in Grade 1 whereas words were read faster than pseudowords in Grade 2 and 3. Analyses on correct response rates corroborated those on efficiency scores. Accuracy increased significantly in Grade 2 (*z* = 5.77, *p* < 0.0001) and Grade 3 (*z* = 7.64, *p* < 0.0001). Lexicality was not significant overall (*z* = 0.52) but lexicality interacted with Grade (Grade 2: *z* = 6.36, *p* < 0.0001; Grade 3: *z* = 8.34, *p* < 0.0001), confirming the presence of a lexicality effect in second and third graders.

**FIGURE 2:**
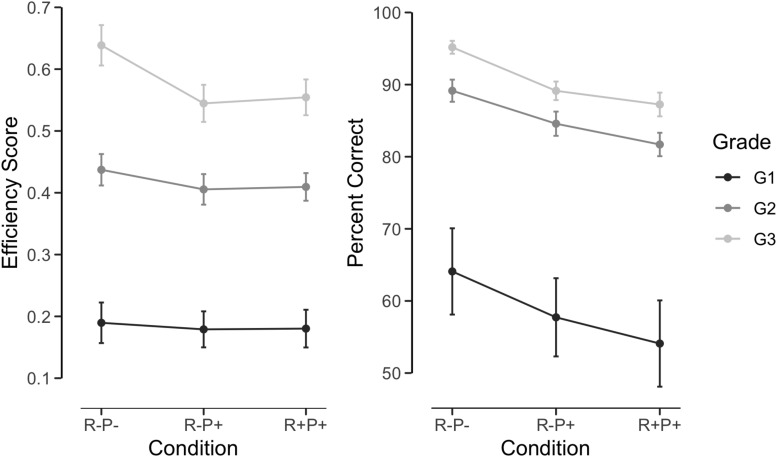
Word reading efficiency and accuracy as a function of root frequency, pattern frequency and grade.

Due to the absence of the R+P− word list, root and pattern frequency effects were assessed separately for words and pseudowords. For words, we performed an analysis of variance with grade as a between-subject factor and condition (R+P+ vs. R−P− vs. R−P+) as a within-subject factor, using contrast analysis to test the effect of root and pattern frequency (see Figure 2). The main condition effect was significant *F*(2,272) = 5.71, *p* = 0.004, $\eta_{\text{p}}^{2}$ = 0.04 with no significant Condition by Grade interaction *F*(4,272) = 2.02, *p* = 0.09, $\eta_{\text{p}}^{2}$ = 0.03. R−P− words were read better than the R−P+ and R+P+ words. In the analysis of correct responses, neither the condition effects nor the interactions between condition and grade approached statistical significance (all *p*s > 0.25).

Thus, although participants obtained better scores on the R−P− words than the R−P+ and R+P+ words, the absence of significant effects in the analysis of correct responses indicates that this effect does not generalize across items. In fact, a few items accounted for a large proportion of errors: two items produced 65% of all errors in the R+P+ condition and two others, 54% in the R−P+ condition. In sum, no clear evidence emerged for an influence of either root or pattern frequency on word reading.

**FIGURE 3:**
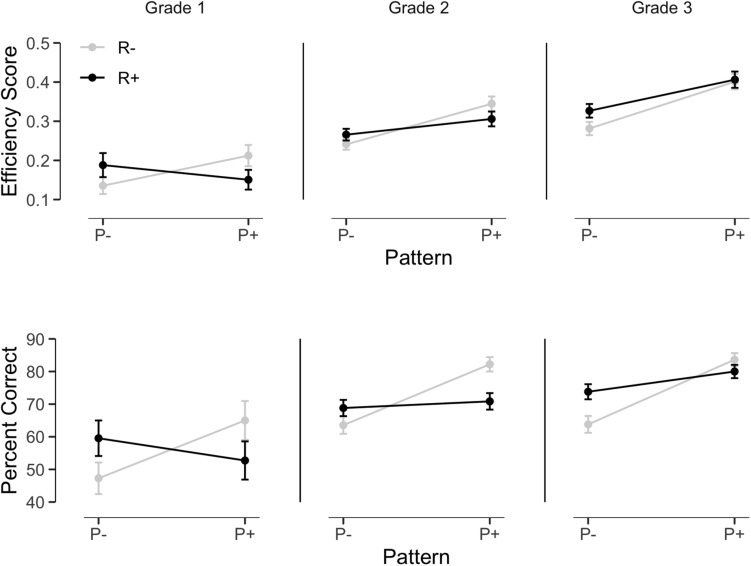
Pseudoword reading efficiency and accuracy as a function of root frequency, pattern frequency and grade.

As can be seen in Figure 3, for pseudowords, a significant Pattern Frequency effect was observed on efficiency scores, *F*(1,136) = 70.48, *p* < 0.001, $\eta_{\text{p}}^{2}$ = 0.34, modulated by Grade, *F*(2,136) = 7.89, *p* < 0.001, $\eta_{\text{p}}^{2}$ = 0.10. The advantage for pseudowords with frequent patterns appeared in Grade 2, *F*(1,58) = 44.78, *p* < 0.001, $\eta_{\text{p}}^{2}$ = 0.44 and Grade 3, *F*(1,57) = 77.04, *p* < 0.001, $\eta_{\text{p}}^{2}$ = 0.57, but was not significant in Grade 1, *F*(1,21) = 2.76, *p* = 0.11, $\eta_{\text{p}}^{2}$ = 0.12. Root frequency was not significant overall, *F* < 1, but the Root frequency by Grade interaction suggested that the influence of root frequency varied across grades, *F*(2,136) = 4.15, *p* = 0.02, $\eta_{\text{p}}^{2}$ = 0.06. Separate analyses by grade indicated a significant advantage for R+ pseudowords in Grade 3 only, *F*(1,57) = 6.21, *p* = 0.02, $\eta_{\text{p}}^{2}$ = 0.10. Finally, the Root by Pattern interaction was also significant, *F*(1,136) = 23.40, *p* < 0.001, $\eta_{\text{p}}^{2}$ = 0.15, the effect of Pattern Frequency being more marked for R− than for R+ pseudowords.

The analyses on correct responses closely paralleled the analysis on efficiency scores. Overall, a 10% Pattern Frequency effect was observed (75.9 vs. 65.3% correct, respectively for P+ and P−) which did not reach significance (*z* = 0.95, *p* = 0.34), but interacted with Grade (*z* = 1.85, *p* = 0.064; *z* = 2.90, *p* < 0.004, respectively for Grade 2 and Grade 3). The Pattern Frequency effect tended to be more marked for pseudowords with rare roots (80.1 vs. 61.1% correct for R−P+ vs. R−P−) than for those with more frequent roots (71.8 vs. 69.4%), as indicated by the Pattern by Root interaction, *z* = −2.18, *p* < 0.03. Neither the main Root Frequency effect nor the interactions with Grade approached significance. Separate analyses by grade indicated that the Pattern Frequency effect was significant in second (*z* = 2.15, *p* = 0.032) and third graders (*z* = 3.22, *p* = 0.001).

Finally, qualitative analyses of errors were performed. In one analysis, errors were categorized as bearing on consonants, on vowels, or on both. A larger proportion of errors was related to vowels than to consonants. For words, 40% of errors concerned vowels and 33% concerned consonants whereas for pseudowords, 64% of errors concerned vowels and 17% consonants. Overall, 77% of errors on vowels were confusions between long and short vowels. Likewise, 51% of errors on consonants in words concerned gemination, which follows the insertion of certain patterns within a root. The distribution of error rates on consonantal roots and predominantly vowel-based word patterns is consistent with the error rates for consonants and vowels. For words and pseudowords, we observed respectively error rates of 19% and 13% on roots against 64% and 75% on patterns.

#### Written Word Comprehension

Word comprehension efficiency improved across grades, *F*(2,136) = 33.02, *p* < 0.001, $\eta_{\text{p}}^{2}$ = 0.33. Mean efficiency scores of 0.22 (SD = 0.09), 0.36 (SD = 0.13) and 0.48 (SD = 0.15) were observed respectively for Grades 1, 2, and 3. The rate of correct response was 85% (SD = 7.17) for Grade 1 and increased to 92.4% (SD = 4.85) and 94.6% (SD = 2.80), respectively, in Grade 2 and 3.

### Morphological Awareness

#### Morpho-Semantic Induction

A mixed model was fitted on correct responses with participants and items as random factors and grade level, type of paradigm (derivational vs. inflectional) and root frequency as predictors. As can be seen from Table 2, a massive difference was observed between derivational and inflectional paradigms (37.3 vs. 84.7% correct), *z* = 7.59, *p* < 0.0001, and performance increased with grade, *z* = 1.62, p = 0.11 for Grade 2, *z* = 4.35, *p* < 0.0001 for Grade 3 relative to Grade 1. Surprisingly, better average performance was observed with less frequent than more frequent roots, although the main root frequency effect did not approach significance, but interacted with Grade 3, *z* = −2.06, *p* = 0.039. Separate analyses per grade level indicated that this reverse root frequency trend only approached significance in Grade 3, *z* = −1.86, *p* = 0.063.

**TABLE 2:** Mean percent correct (SD in brackets) in the morpho-semantic induction task per condition and grade.

Type of morphology	Root frequency	Grade 1	Grade 2	Grade 3
Derivational	R+	25.76 (16.58)	33.76 (20.08)	37.79 (20.31)
	R−	30.81 (17.79)	36.44 (16.94)	49.71 (22.94)
	Average	28.28	35.10	43.75
Inflectional	R+	77.27 (16.96)	83.76 (11.93)	85.92 (11.81)
	R−	79.80 (13.99)	80.65 (12.61)	91.52 (10.18)
	Average	78.54	82.20	88.72

*R+, frequent root; R−, rare/non-existent root.*

Third graders’ errors included more lexicalizations on R+ items than on R− items for both derivational (R+: 29.6%, R−: 9.4%) and inflectional (R+: 42.8%, R−: 11.8%) paradigms. We also found that the root was often preserved and errors mostly affected patterns (only 20% of lexicalization errors concerned the root and 65% the pattern). Many errors were words formed from the combination of the root with a pattern different from that of the stimulus. For example, to the stimulus/barika/, children replied with the word بِركة/birkat/*lake* instead of the pseudoword expected مَبرَك/mabrak/whose pattern designates a place. This word is constructed from the three {brk} consonants of the stimulus and the pattern {CiCCat}. When those errors were discarded, the number of errors for R+ (derivational: 304, inflectional: 56) and R− (derivational: 316, inflectional: 52) conditions closely converged.

#### Morpho-Phonological Induction

**FIGURE 4:**
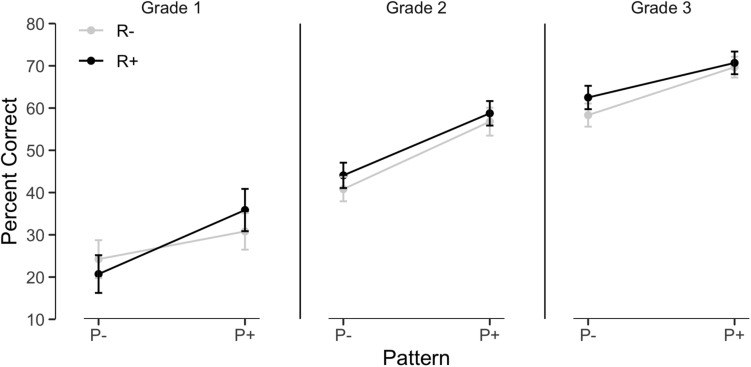
Morpho-phonological induction accuracy as a function of root frequency, pattern frequency and grade.

A mixed model was fitted on correct responses with participants and items as random factors and grade level, root and pattern frequency as predictors. The analysis indicated significant improvements across grades, *z* = 4.67, *p* < 0.0001 and z = 7.86, *p* < 0.0001, respectively, for Grade 2 and 3 relative to Grade 1. Mean percentage correct raised from 27.9% in Grade 1 to 50.1% in Grade 2 and 65.3% in Grade 3 (see Figure 4). Interestingly, despite the improvements, the main effect of Pattern Frequency was significant, *z* = 3.52, *p* < 0.0005 and stable across age levels, as shown by the lack of interaction with Grade. The Root Frequency effect did not reach significance, *z* = 0.33, and did not vary with grade. Again, errors were more frequent on vowels (46%) than on consonants (16%) or both (38%). Separate models indicated that the pattern frequency effect was significant at each grade level, without any other significant effect.

### Regression Analyses

The aim of this section is to assess the association between reading and MA, controlling for general cognitive factors as well as for phonological abilities. To that end, we first examined the relationships between reading performance and other abilities. Ten predictors were considered: Non-verbal intelligence (RAVEN), receptive vocabulary (VOCAB), working memory (forward and backward correct responses), rapid naming (RAN), phonemic discrimination (DISCR), letter naming (GRAPH, efficiency scores), phoneme deletion (DELET, average of the three phoneme deletion conditions correct responses), morpho-semantic induction (MORPHS, total number of correct responses), and morpho-phonological induction (MORPHP, total number of correct responses).

Table 3 displays the intercorrelations among predictors. Despite the relatively wide age range considered, predictors are only moderately correlated with each other. Unsurprisingly, the strongest pairwise correlation is between the two morphological tests (*r* = 0.53). Interestingly, the morpho-phonological induction (*MORPHP*) test displays stronger associations with the other phonological manipulation assessment (Phoneme Deletion, *DELET*, *r* = 0.46) than the morpho-semantic induction test (*MORPHS*, *r* = 0.20). This configuration suggests that both induction techniques may tap into partly distinct aspects of MA, the phonological patterning on one hand, and the semantic relation on the other.

**TABLE 3:** Intercorrelations among predictor variables, and correlations with word reading efficiency, pseudoword reading efficiency, and word comprehension efficiency.

	RAVEN	VOCAB	SPAN Fwd	SPAN Bwd	RAN	DISCR	GRAPH	DELET	MORPHP	MORPHS
RAVEN	–									
VOCAB	0.14	–								
SPAN Fwd	0.01	0.08	–							
SPAN Bwd	0.22*	0.12	0.32***	–						
RAN	0.08	0.23**	0.20*	0.18*	–					
DISCR	0.06	0.15	0.10	0.24**	0.35***	–				
GRAPH	0.07	0.25**	–0.07	0.04	0.14	0.04	–			
DELET	0.28***	0.07	0.12	0.16	0.08	0.19*	0.12	–		
MORPHP	0.22**	0.34***	0.16	0.15	0.24**	0.19*	0.25**	0.46***	–	
MORPHS	0.19*	0.28***	0.14	0.09	0.25**	0.19*	0.18*	0.20*	0.53***	
W Eff	0.18*	0.34***	0.09	0.19*	0.23**	0.16	0.57***	0.33***	0.60***	0.43***
PW Eff	0.20*	0.22**	0.09	0.22**	0.19*	0.15	0.50***	0.42***	0.54***	0.34***
W Comp	0.16*	0.31***	0.12	0.22*	0.19*	0.19*	0.45***	0.54***	0.36***	0.35***

*p < 0.05, **p < 0.01, ***p < 0.001, VOCAB, vocabulary; SPAN Fwd, span forward; SPAN Bwd, span backward; RAN, rapid naming; DISCR, phoneme discrimination; GRAPH, letter naming; DELET, phoneme deletion; MORPHP, morpho-phonological induction; MORPHS, morpho-semantic induction; W Eff, word efficiency; PW Eff, pseudoword efficiency; W Comp, word comprehension.

Table 3 also displays the raw pairwise correlations between the ten predictors and the three measures of reading (word and pseudoword oral reading efficiency, word comprehension). All three dependent measures show high correlations with grapheme knowledge, phoneme deletion, as well as with the two MA tasks. Word reading and word comprehension also correlate strongly with vocabulary.

In order to determine the extent to which the different predictors account for the ability to read and understand written words, stepwise regression analyses were conducted with word and pseudoword oral reading efficiency and word comprehension as dependent variables. The set of predictors included grade, Raven, vocabulary, forward and backward digit spans, phoneme discrimination, rapid naming efficiency, letter naming efficiency, consonant deletion, and morpho-semantic and morpho-phonological induction scores. General and phonological predictors (Grade, Raven, Vocabulary, Spans, Rapid naming, and phoneme discrimination) were entered into the regression as a first forced step. Then, the stepwise procedure was used to test the contribution of letter knowledge, consonant deletion, and the two morphological induction tests.

The final models appear in Table 4. For each dependent measure, the regression model accounted for a large proportion of variance, respectively 61%, 50%, and 50% for word reading, pseudoword reading, and word comprehension, and the morpho-phonological test performance contributed significantly in each of the three case.

**TABLE 4:** Stepwise regression analyses: final models.

	Unstandardized	Standard error	Standardized	*t*	*p*
**Word reading**					
(Intercept)	–0.2500	0.2000		–1.24	0.220
Grade	0.1000	0.0200	0.330	4.36	<0.001
DISCR	0.0008	0.0020	0.030	0.43	0.670
RAVEN	–0.0006	0.0028	–0.010	–0.22	0.830
VOCAB	–0.0027	0.0035	–0.050	–0.77	0.440
SPAN Fwd	–0.0026	0.0080	–0.020	–0.33	0.740
SPAN Bwd	0.0086	0.0091	0.060	0.94	0.350
RAN	0.0900	0.0700	0.080	1.28	0.200
GRAPH	0.2500	0.0400	0.410	6.95	<0.001
MORPHP	0.0029	0.0007	0.300	4.25	<0.001
**Pseudoword reading**					
(Intercept)	–0.0600	0.1400		–0.46	0.650
Grade	0.0400	0.0200	0.210	2.44	0.020
RAVEN	–0.0002	0.0019	–0.005	–0.08	0.940
VOCAB	–0.0029	0.0023	–0.090	–1.23	0.220
SPAN Fwd	–0.0019	0.0054	–0.020	–0.35	0.720
SPAN Bwd	0.0090	0.0061	0.100	1.46	0.150
RAN	0.0400	0.0500	0.070	0.97	0.330
DISCR	–0.0001	0.0013	–0.007	–0.11	0.910
GRAPH	0.1300	0.0200	0.360	5.38	<0.001
DELET	0.0200	0.0056	0.210	2.86	0.005
MORPHP	0.0013	0.0005	0.230	2.66	0.009
**Word comprehension**					
(Intercept)	–0.2000	0.1700		–1.17	0.250
Grade	0.0800	0.0200	0.370	4.21	0.000
RAVEN	–0.0018	0.0024	–0.050	–0.75	0.460
VOCAB	–0.0016	0.0029	–0.040	–0.55	0.590
SPAN Fwd	–0.0006	0.0067	–0.006	–0.09	0.930
SPAN Bwd	0.0078	0.0076	0.070	1.03	0.310
RAN	0.0400	0.0600	0.050	0.77	0.440
DISCR	0.0014	0.0016	0.060	0.82	0.410
GRAPH	0.1300	0.0300	0.290	4.25	<0.001
DELET	0.0200	0.0069	0.160	2.18	0.030
MORPHP	0.0013	0.0006	0.170	1.99	0.050

*DISCR, phoneme discrimination; VOCAB, vocabulary; SPAN Fwd, span forward; SPAN Bwd, span backward; RAN, rapid naming; GRAPH, letter naming; MORPHP, morpho-phonological induction.*

For word reading, two significant predictors, grapheme knowledge and morpho-phonological induction contributed to the final equation, accounting together for 22% of variance, over and above the forced predictors. For the word comprehension task, the same two predictors as well as phoneme deletion were significant, and the three variables accounted for 15% of the variance. Similarly, for pseudoword reading, the three same predictors accounted for 24% of additional variance.

In addition, we ran additional regression analyses in which the order of entry was constrained. General predictors (Grade, Raven, Vocabulary, Spans, Rapid naming, and phoneme discrimination) were entered in a first block, and phoneme deletion and letter naming were entered in the second block. To assess whether the MA tasks account for a significant additional portion of variance, we then entered successively morpho-phonological and morpho-semantic induction performance in a third and fourth block, or vice-versa. The main results appear in Table 5.

**TABLE 5:** Hierarchical regression analyses.

	Block 1	Block 2		Block 3		Block 4		Block 3		Block 4	
	General predictors	Phoneme deletion and letter naming		Morpho-phonological induction		Morpho-semantic induction		Morpho-semantic induction		Morpho-phonological induction	
	Δ	Δ	*p*	Δ	*p*	Δ	*p*	Δ	*p*	Δ	*p*
Word efficiency	39.3	19.3	<0.001	3.34	0.001	0.70	0.13	2.03	0.004	2.01	0.01
Pseudoword efficiency	25.5	21.6	<0.001	2.80	0.009	0.19	0.49	0.99	0.12	2.0	0.03
Word comprehension	35.4	13.0	<0.001	1.56	0.05	<0.01	0.66	0.49	0.27	1.15	0.09

*Δ indicates the percentage of additional variance accounted for by a given predictor or set of predictors.*

As can be seen, for all three dependent measures, the contribution of the morpho-phonological induction test was significant over and above general predictors, phoneme deletion and letter naming, and accounted for an additional 1.5 to 3.3% of explained variance. Moreover, for the word and pseudoword reading measures, performance in the morpho-phonological test was even predictive after the morpho-semantic score was entered. Conversely, the morpho-semantic induction test had a significant contribution only on word efficiency, and did not significantly add to the model after morpho-phonological induction was entered. In sum, the regression analyses converge to demonstrate that the morpho-phonological induction test accounts for some unique variance in reading performance.

## Discussion

The main aim of our study was to investigate the influence of morphological structure on the early stages of reading acquisition, and more specifically, the role of root and pattern units in beginners from Grade 1 to 3. To that end, we assessed the presence of effects of root and word pattern frequency on oral reading as well as in morphological manipulations. In addition, we examined the links between phonological and morphological abilities and word recognition abilities. Overall, the results show that from Grade 2 onward reading performance is sensitive to pattern frequency but not to root frequency. The results of the morpho-phonological oral induction test provide additional support to that conclusion. Furthermore, regression analyses indicate that the latter test is predictive of word and pseudoword oral reading as well as word comprehension, over and above the contribution of other factors. We first discuss early MA and its links with reading acquisition, and second, the direct evidence of morphological sensitivity in word and pseudoword processing.

### Morphological Awareness and Reading Ability

One purpose of our study was to examine the relation of MA to word reading, with new oral induction tasks which we assumed would provide purer measures of explicit morphological processing. We devised two tests, a morpho-semantic induction task analogous to Berko’s (1958) famous Wug test, and a similar morpho-phonological induction task devoid of semantic support, which focuses more specifically on the phonological dimension of morphological paradigms. We first analyze the results of these two tasks and then discuss their relation to reading ability.

In the morpho-semantic oral induction test, inflectional patterns produced significantly better scores than derivational ones. The large difference between derivational and inflectional paradigms could be taken to be an effect of pattern familiarity, as inflectional patterns are much more familiar. However, this difference could also be due to explicit teaching of some of the inflectional patterns at school, or to differences in the complexity of the phonological modifications. By contrast, no clear root effect was observed, but responses indicated a trend to a reverse root effect, which was significant in third grade in the analysis per subject only. An analysis of erroneous responses suggested a potential explanation for this unexpected result. With frequent roots, children often produced lexical forms by preserving the root but combining it with a pattern different from the one required by the induction rule. When those errors were discarded, success rates in the R+ and R− series were very similar. Thus, the results from the morpho-semantic induction test suggest that third graders identified frequent roots and often produced lexical candidates based on these roots rather than applying the required manipulation rule which leads to a pseudoword response. It seems likely that children took the semantic information conveyed by the root into account but somehow neglected the sentence context deemed to induce the targeted change in pattern. Although contrary to our expectations at first glance, these results suggest some degree of sensitivity to the semantics of roots.

Clearer evidence of sensitivity to morphological structure was provided in the morpho-phonological oral induction test. In addition to large global performance gains across age groups, analyses of correct response rates by subject and by item indicated a facilitation effect of pattern frequency, which was already present in Grade 1. In contrast, no evidence of an influence of root frequency was observed. The morpho-phonological induction task is similar to some phonological awareness tests in which children are trained by examples to perform a systematic transformation such as consonant deletion for instance (Content et al., 1986), although the transformations required in the present task were much more complex and combined several phonological operations. Thus, the task is likely to tap into explicit phonological manipulation abilities. However, the observation that performance improves when the change concerns more frequent patterns provides clear evidence that children benefit from morphological knowledge over and above their phonological manipulation skills.

In sum, the morphological induction tasks provide evidence of an early sensitivity to the morphological structure of spoken words. In the morpho-semantic induction situation, first-graders already succeeded to some extent to generalize the demonstrated morphological manipulation to new pseudowords, and performance was much higher with simpler and more familiar inflectional rules that mostly concern the word pattern. Similarly, in the morpho-phonological test, first-graders’ correct responses were impacted by pattern frequency. The early sensitivity to word patterns in the oral modality fits well with Saiegh-Haddad and Taha (2017) findings based on morphological relatedness judgments, which showed a large advantage for word-pattern relations over root relations in first- to fourth-graders. The observation of a pattern advantage in the present tasks in which all manipulations were based on pseudowords further confirms that this sensitivity is really morphological and not determined by word knowledge. The new induction tasks that we have designed appear to provide an elegant and valid technique to assess children’s awareness of morphological relations while avoiding the potential confound of vocabulary knowledge.

Given the observation of early sensitivity to morphology in the oral induction tasks, it is of interest to examine whether there is an association between performance in MA tasks and visual word recognition. The pairwise correlations between predictors and the three dependent measures, i.e., word reading, pseudoword reading and word comprehension, showed positive correlations with the two MA tasks as well as with Raven IQ, vocabulary, backward span, rapid naming, grapheme knowledge, and phoneme deletion. More importantly, stepwise regression analyses revealed two significant predictors for word reading, grapheme knowledge and morpho-phonological induction, over and above the effect of grade level. The same predictors as well as phoneme deletion were significant for pseudoword reading. Similarly, significant contributions of grapheme knowledge, consonant deletion and morpho-phonological induction to word reading comprehension were observed.

Because the frequency of morphemes was manipulated in the oral reading task, one might wonder if the nature of the task might have induced positive correlations with MA. It is worth noting that the level of performance observed in the present study is similar to other studies in Arabic which did not focus on morphology. For example, in Tibi and Kirby’s (2017) study, third graders achieved 78% success in pseudoword reading, a result that meets the scores for our participants at the same school level (75%; see also Elbeheri and Everatt, 2007). Indeed, in Arabic, the vast majority of words are at least bimorphemic. Consequently, the words in the present study were typical of Arabic language structure and the pseudowords had a structure similar to words and representative of the Arabic linguistic characteristics. Furthermore, the correlation with morpho-phonological awareness was not limited to word and pseudoword oral reading. The results showed a similar contribution of morpho-phonological induction to word comprehension, in which no morphological constraints affected the selection of materials, confirming the conclusion that the association between reading performance and MA cannot be explained by the characteristics of the items in the oral reading tasks.

As in other Arabic countries, MSA, which is used in conventional writing, differs from the spoken Arabic used in everyday life in Lebanon. Although the standard and spoken Arabic languages share many phonological, morphological, lexical and syntactic features, spoken languages vary in their linguistic distance to MSA (Saiegh-Haddad, 2003; Holes, 2004). In this study, frequent patterns in MA tasks were chosen among those common to both Arabic registers, sometimes with minimal phonetic variations (e.g., faʕʕāl in MSA produced feʕʕēl in Lebanese spoken Arabic). Therefore, results for MA cannot be generalized to morphemic constituents that are specific to MSA because MA is “more a language- or variety-specific construct” (Schiff and Saiegh-Haddad, 2017). However, MSA and spoken Arabic share the general principles of morphological organization, with consonantal roots and patterns, so that sensitivity to morphology might possibly transfer from the domain of speech to the domain of literacy.

In summary, the morpho-phonological induction performance came out as a significant predictor for all three measures of reading ability, over and above the effect of grade level, grapheme knowledge, phonemic awareness and other potential predictors, thus indicating a unique association of MA to printed word recognition. The manipulations required by the morphological induction tasks necessarily involve phonological processing. More generally, in Arabic, morphological manipulations require sufficiently developed phonological skills as the phonological and morphological structures are extremely tightly related (Taha and Saiegh-Haddad, 2017). The association between these two abilities could explain the strong correlation observed between the morpho-phonological induction task and reading performance. These findings thus reinforce the conclusion that there is a link, possibly causal, between MA and reading acquisition in Arabic. They concur with previous evidence of a contribution of MA to word reading (Abu-Rabia, 2007; Saiegh-Haddad and Geva, 2008; Boukadida et al., 2009; Abu-Ahmad et al., 2014; Tibi et al., 2018; Tibi and Kirby, 2019), pseudoword reading (Tibi and Kirby, 2017, 2019; Tibi et al., 2018) and reading comprehension (Abu-Rabia, 2007; Asadi et al., 2017c; Layes et al., 2017; Tibi et al., 2018; Tibi and Kirby, 2019). Taken together those findings underscore the role of morphological structure as an important component of Arabic reading development as proposed by Saiegh-Haddad (2018) in her model of Arabic reading acquisition.

### Sensitivity to Morpheme Frequency in Word Recognition

The second main objective of the present study was to examine whether beginning readers are sensitive to the frequency of root and patterns in reading aloud. In summary, the results revealed three major reliable findings: an advantage for word reading over pseudoword reading in second and third graders; no evidence of an effect of either root or pattern frequency on word reading; a facilitatory effect of pattern frequency on pseudoword reading, in Grade 2 and 3.

One contrary finding was reported by Shalhoub-Awwad and Leikin (2016) in a lexical decision task with cross-modal root priming, who observed significant effects already in second graders, suggesting that sensitivity to morphological structure may already be present at that stage. Interestingly, the priming effect was larger in second graders than in fifth graders, perhaps due to faster access to the full orthographic form than to the morphological components in the elder participants. However, the authors provided no information about the frequency of the target items or their familiarity for beginning readers. Furthermore, as there was no semantic priming condition, it is unclear whether the facilitation effect was due to the morphological or to the semantic relation between primes and targets. In the present task, words were of medium to high frequency. They were highly familiar to the children and could already be present in their mental lexicons, so that no influence of constituents frequency would be obtained.

The absence of effect of morphological constituents’ frequency in the word reading task appears incompatible with the hypothesis of obligatory morphological decomposition proposed by Boudelaa and Marslen-Wilson (e.g., Boudelaa and Marslen-Wilson, 2011, 2015; Boudelaa, 2014). However, the latter view is based on data from expert readers with unvowelled print, and we speculate that the influence of morphology could be more important with unvowelled than with full vowelled orthography. As suggested by several authors (e.g., Hansen, 2014), unvowelled text processing is likely to resort to lexical/orthographic knowledge to a larger extent. In fact, the absence of morpheme frequency effects for words in the present study is reminiscent of similar findings with adult expert readers. Abu-Rabia and Awwad (2004) reported no morpheme frequency effects for high-frequency words and Grainger et al. (2003) found that the effect of root frequency was larger and only significant for low-frequency words. Indeed, several authors claimed that parsing a word into morphemes may involve benefits mainly for low-frequency words. When lexical reading is possible because the whole word is represented in the mental lexicon, the cost induced by a morphological reading strategy may overcome the benefits, and the whole word lexical process might be faster and more reliable (Schreuder and Baayen, 1995; Traficante and Burani, 2003; Burani, 2010; Angelelli et al., 2014).

Contrary to the findings from word reading, a clear influence of morphological composition was obtained for pseudoword reading in Grade 2 and 3. The morphological facilitation obtained in pseudoword reading was limited to patterns. This finding is in line with the results from the morphological induction tasks, which also indicated sensitivity to patterns only, as well as with Bar-On et al.’s (2018) study, which showed that already in Grade 2 children were able to correctly add vocalic patterns when they read unvowelled pseudowords with morphological structure (see also Shalhoub-Awwad, 2019).

Several hypotheses may explain why the results indicate evidence of pattern frequency only. One possible explanation of the differential sensitivity to consonantal roots and vocalic patterns is that consonant processing is easier than vowel processing. Both in pseudoword reading and in the morpho-phonological induction task, there were many more errors on vowels than on consonants, leading to better preservation of the root. Thus, the easier processing of consonants may have masked an effect of root frequency. In contrast, more errors occur on vowels, so that pattern frequency may be more helpful.

Moreover, although root frequency does not seem to affect accuracy, it might have an influence on processing which cannot be detected in the naming task. Oral reading cannot be completed until the entire phonological information is available, and therefore requires the decoding of both the root as well as the pattern. Hence, even if the root is extracted faster than the pattern, due to differences between consonant and vowel processing, it will be necessary to wait until the pattern is available to produce a response. Decision tasks might thus be more appropriate to uncover root frequency effects because root identification is sufficient for word perception but not for production, which requires a complete phonological specification (see Deutsch and Malinovitch, 2016; Bar-on et al., 2018 for a similar argument).

According to this view, the difference between consonants and vowels could stem from phonetic and phonological characteristics. Short and long vowels mainly differ in terms of duration, and may thus be harder to discriminate in context, whereas the distinctive properties of consonants might be more salient. More generally, it has been argued that consonants and vowels play different roles in language processing. Consonants typically provide information mostly relevant to word identification, whereas vowels mostly support rhythm and prosody and convey morphological and syntactic cues. While such a distribution of function may be universal (Nespor et al., 2003), the distinction is particularly salient in Semitic languages. Consonants, as specification of the roots, concern the lexicon and inform about meaning, whereas vowels mostly provide morphosyntactic information. Indeed, Boudelaa (2015) argued from cross-modal priming experiments with adult Arabic speakers that consonants and vowels are processed separately, and that consonant information is specifically used to generate lexical hypotheses. Regarding written language processing, Berent and Perfetti (1995) argued that in adults, phonological conversion occurs separately for vowels and consonants, with consonants being transcoded in a first cycle and faster than vowels. Although further studies questioned whether the distinction was general, or specific to English orthography, recent studies suggest that the consonant skeleton may play an early role in access to phonology (Duñabeitia and Carreiras, 2011; Perea et al., 2018), and that the organization of consonant and vowel letters determines perceptual units of print (Chetail and Content, 2012, 2014, 2017; Chetail et al., 2014, 2018).

In sum, the absence of a root frequency effect should not be taken to conclude that children are insensitive to root properties. In fact, it would seem hard to explain how they could isolate patterns without at the same time extracting the roots. As Ravid and Schiff (2006, p. 809) argued, “the ability to manipulate roots by definition also involves the manipulation of patterns, since it is the combination with the pattern that gives the phonological shape to the word.” Similarly, the sensitivity to the frequency of the pattern necessarily implies the isolation of the root. Furthermore, the pattern frequency effect was modulated by root frequency. The pattern facilitation was smaller for frequent roots than for less frequent/non-existent ones, thus indicating that root frequency does actually influence pseudoword reading. One likely explanation of this interaction is items containing frequent roots activate lexical candidates which might interfere with the correct response.

Regarding the developmental course of abilities, the first indications of morphological decomposition observed in the present study are contemporary with the apparition of the lexicality effect, suggestive of a lexical reading procedure. Thus, our findings are compatible with a developmental scenario according to which first graders decode pseudowords through GPCs, and gradually acquire representations of words and morphological constituents as shown by the lexicality and morphology effects in Grade 2. The high level of accuracy in pseudoword reading observed already in Grade 1 attests to a good mastery of phonological recoding. Presumably, this would allow the beginning readers to attend to larger orthographic units, as proposed by the grain-size hypothesis (Ziegler and Goswami, 2005). The indications of morphological sensitivity in oral reading and the association of reading performance with MA suggest that morphological structure contributes to the constitution of larger orthographic units. With orthographic and morpho-orthographic processing mechanisms, children will progressively abandon the use of phonological information conveyed by short vowels (Bar-On and Ravid, 2011; Saiegh-Haddad and Schiff, 2016; Schiff and Saiegh-Haddad, 2017) and thus shift to unvowelled orthography. A similar development in which reading relies more on morphemic constituents and less on graphemes and analytical sublexical processing has been shown in Italian (Burani et al., 2008), a shallow orthography. Our results converge with others (Ravid, 1996; Bar-On and Ravid, 2011; Schiff et al., 2012; Saiegh-Hadddad, 2013; Saiegh-Haddad and Schiff, 2016; Schiff and Saiegh-Haddad, 2017; Taha and Saiegh-Haddad, 2017; Bar-on et al., 2018) to demonstrate that such an evolution also occurs in Semitic writing systems in which morphemic constituents are spatially distributed within letter strings and correspond to non-adjacent letters.

In the recent years, several authors have proposed theoretical models to integrate the contribution of morphology to reading acquisition. For Semitic languages, Saiegh-Haddad (2018) introduced MAWRID, a Model of Arabic Word Reading In Development, and Share and Bar-On (2018) presented the triplex model of Hebrew reading development. Saiegh-Haddad (2018) argues that the early grapheme–phoneme conversion mechanism is rapidly augmented by a morpho-orthographic decoding procedure appearing around the second grade. Share and Bar-On (2018) envisage a progression from phonological grapheme–phoneme conversion (Phase 1, Grade 1) to lexical and morpho-orthographic processing (Phase 2, Grade 2). Our results fit well with both descriptions.

## Conclusion

The present study provides clear evidence for the early emergence of a sensitivity to the frequency of patterns in both pseudoword reading and oral morpho-phonological manipulations. This supports the view that because “in Arabic, morphological structure provides a domain of knowledge that is exceptionally consistent and regular both in terms of linguistic form and linguistic meaning” (Boudelaa and Marslen-Wilson, 2015, p. 24), it naturally induces the decomposition of words into their constituent morphemes. Despite the non-concatenative nature of morphological units and the transparency of the orthography, young readers take advantage of the frequency of morphological constituents. The present findings support the view that morphology, besides phonology, plays a role in reading unfamiliar words, and corroborate the importance of MA, besides phonological awareness, in reading acquisition in Arabic.

## Data Availability Statement

The datasets presented in this study can be found at the following link: https://osf.io/f2ycj/.

## Ethics Statement

The studies involving human participants were reviewed and approved by Ethics Committee of the Faculty of Psychological Sciences of the Université libre de Bruxelles. Written informed consent to participate in this study was provided by the participants’ legal guardian/next of kin.

## Author Contributions

AC and CE designed the study, analyzed the data, and drafted the manuscript. CE collected the data. Both authors contributed to the article and approved the submitted version.

## Conflict of Interest

The authors declare that the research was conducted in the absence of any commercial or financial relationships that could be construed as a potential conflict of interest.

## Footnotes

1 Root and patterns are noted within curly brackets.

2 In the representation of word patterns, the letter C is used to indicate the consonants of the root.

3 The smaller number of participants in Grade 1 is due to the last-minute withdrawal of two of the three schools.
