# Supplementary material for: Early Sensitivity to Morphology in Beginning Readers of Arabic
Source: Front Psychol. 2020 Sep 23;11:552315. doi: 10.3389/fpsyg.2020.552315 (PMC7538675; doi:10.3389/fpsyg.2020.552315)
Supplement: Supplementary file 1 [file Table_1.pdf]

1. Psycholinguistic characteristics of the reading lists

	Number of letters	Number of phonemes	Proportion of CVC syllables	Familiarity Rating	Root Frequency[1]	Pattern Frequency[1]
**Word R+P+**	4.3	5.4	0.80	2.99	2.58	3.97
**Word R-P+**	4.0	5.4	0.70	2.94	0.94 (n=8)	3.41
**Word R-P-**	4.5	5.6	0.75	2.95	— [2]	— [2]
**PW R+P+**	4.3	5.5	0.70	–	2.78	3.57
**PW R+P-**	4.7	5.5	0.75	–	2.77	— [2]
**PW R-P-**	4.4	5.4	0.75	–	— [2]	— [2]
**PW R-P+**	4.4	5.5	0.75	–	— [2]	3.30

[1] *Based on log-transformed frequency from Aralex.*

[2] *No average computed since most roots/patterns are inexistent*

2. Results for control variables

2.1 Letter naming. The correct response rate was already high in Grade 1 (m = 79.6%, SD = 12.4), and performance was close to ceiling in Grades 2 and 3 (respectively m = 94.2%, SD = 4.6, m = 94.8%, SD = 4.0). An analysis of variance on efficiency scores (ES) by grade showed a significant effect, $F(2, 136) = 9.36$, $p < .001$, $\eta^2_p = .12$ due to the difference between 1st graders (ES = 0.73, SD = 0.34) and the two older groups (respectively ES = 1.06, SD = 0.36 et ES = 1.07, SD = 0.31).

2.2 Phoneme discrimination. The ANOVA on correct responses showed a significant effect of Grade, $F(2, 135) = 4.44$, $p = .01$, $\eta^2_p = .06$, and of type of pair, $F(1, 135) = 210.61$, $p < .001$, $\eta^2_p = .61$, due to the large difference between same and different trials (96.4 vs 78.2% respectively). Performance was slightly weaker in second graders (85.4%) than in the younger (88.6%) or older (88.8%) groups. Errors mainly concerned emphatic/non-emphatic pairs with only 58% correct overall.

2.3 Phoneme deletion. Results showed high success rates in the three conditions at all grade levels. The ANOVA with Grade and Condition indicated a significant difference amongst grades, $F(2, 136) = 4.62$, $p =.01$, $\eta^2_p =.06$, due to the improvement between Grade 1 (70.3% overall, SD = 23.0) and Grades 2 and 3 (81.4%, SD = 15.2 and 82.6%, SD = 15.4). There were significant differences across conditions, $F(2, 272) = 15.10$, $p < .001$, $\eta^2_p = .10$. Performance was best in the condition involving deletion of the initial consonant before a long vowel (87.2%, SD = 19.2), and worst for deletion of the initial consonant before a short vowel (73.9%, SD = 2.69). Deletion of the pre-final consonant was intermediate (79.4%, SD = 2.02).

2.4 Rapid Naming. The ANOVA revealed a large difference across conditions (0.82 vs 1.08 correct, respectively for the varied and the repeated condition), $F(1, 136) = 125.16$, $p < .001$, $\eta^2_p = .48$ but there was no indication of a developmental change, $F < 1$.

3. Appendix

3.1 Word and pseudoword reading lists

**Words R+P+**	Phonology	Meaning	Familiarity	Root	Root Frequency[1]	Pattern	Pattern Frequency[1]	Structure
طَعام	/ṭaʕām/	food	3.68	/ṭʕm/	2.04	/CaCāC/	4.09	Cv.CVC
عالِم	/ʕālim/	scientist	2.45	/ʕlm/	3.73	/CāCiC/	4.57	CV.CvC
عَصير	/ʕaṣīr/	juice	3.27	/ʕṣr/	2.70	/CaCīC/	4.46	Cv.CVC
تَدْريس	/tadrīs/	teaching	2.95	/drs/	3.04	/taCCīC/	2.47	CvC.CVC
حَلّاق	/ħallāq/	hairdresser	2.77	/ħlq/	2.10	/CaCCāC/	3.42	CvC.CVC
مَسْحور	/masħūr/	fascinated	1.86	/sħr/	1.87	/maCCūC/	3.97	CvC.CVC
طَبيب	/ṭabīb/	doctor	3.50	/ṭbb/	2.52	/CaCīC/	4.46	Cv.CVC
مَزْروع	/mazrūʕ/	planted	2.64	/zrʕ/	2.54	/maCCūC/	3.97	CvC.CVC
مَلْعَب	/malʕab/	yard	3.55	/lʕb/	2.89	/maCCaC/	3.80	CvC.CvC
رَفيق	/rafīq/	friend	3.23	/rfq/	2.41	/CaCīC/	4.46	CvC.CVC

**Words R-P-**	Phonology	Meaning	Familiarity	Root	Root Frequency[1]	Pattern	Pattern Frequency[1]	Structure
ريشة	/rīʃat/	feather	2.82	no root		no pattern		CV.CvC
ساحَة	/sāħat/	square	2.55	no root		no pattern		CV.CvC
فَتاة	/fatāt/	girl	3.55	no root		no pattern		Cv.CVC
فِسْتُق	/fustuq/	nut	2.18	/fstq/	-0.02	/CuCCuC/	0.44	CvC.CvC
دولاب	/dūlāb/	wheel	2.95	no root		no pattern		CV.CvC
صُنْدوق	/ṣundūq/	box	2.90	no root		no pattern		CV.CVC
بُسْتان	/bustān/	orchard	3.24	/bstn/	-1.52	/CuCCāC/	1.94	CvC.CVC
عُصْفور	/ʕuṣfūr/	bird	3.59	no root		no pattern		CvC.CVC
صَرْصُور	/ṣarṣūr/	cockroach	2.73	no root		no pattern		CvC.CVC
بَطّيخ	/baṭṭīX/	watermelon	2.95	no root		no pattern		CvC.CVC

**Words R-P+**	Phonology	Meaning	Familiarity	Root	Root Frequency[1]	Pattern	Pattern Frequency[1]	Structure
دُرّاق	/durrāq/	peach	2.59	/drq/	-0.14	/CuCCāC/	1.98	CvC.CVC
أَرْنَب	/ʔarnab/	rabbit	3.14	/rnb	0.37	/ʔaCCaC/	4.04	CvC.CvC
غُرْفَة	/ɣurfat/	room	3.41	/ɣrf/	0.72	/CuCCat/	1.86	CvC.CvC
قَضيب	/qaḍīb/	stick	1.62	/qḍb/	0.83	/CaCīC/	4.46	Cv.CVC
نَحْلَة	/naħlat/	bee	3.23	/nħl/	0.94	/CaCCat/	2.68	CvC.CvC
دَجاج	/daʒāʒ/	chicken	3.55	/dʒʒ/	1.05	/CaCāC/	4.09	Cv.CVC
قَميص	/qamīs/	shirt	2.90	/qms/	1.12	/CaCC/	4.46	Cv.CVC
زُجاج	/zuʒāʒ/	glass	3.18	/zʒʒ/	1.58	/CuCāC/	3.38	Cv.CVC
سرير	/sarīr/	bed	3.41	no root		/CaCīC/	4.46	Cv.CVC
قرية	/qarjat/	village	2.32	no root		/CaCCat/	2.68	CvC.CvC

**Pseudowords R+P+**	Phonology	Root	Root Frequency	Pattern	Pattern Frequency	Structure
طَليب	/ṭalīb/	/ṭlb/	3.33	/CaCīC/	4.46	Cv.CVC
طابِخ	/ṭābiX/	/ṭbX/	1.32	/CāCiC/	4.57	CV.CvC
وِقاف	/wiqāf/	/wqf/	3.30	/CiCāC/	4.15	Cv.CVC
مَشْهَر	/maʃhar/	/ʃhr/	2.95	/maCCaC/	1.93	Cv.CVC
وَجيد	/waʒīd/	/wʒd/	3.15	/CaCīC/	4.46	Cv.CVC
كَتْبَة	/katbat/	/ktbt/	3.55	/CaCCaC/	2.68	CvC.CvC
غِسال	/ɣisāl/	/ɣsl/	1.47	/CiCāC/	4.15	Cv.CVC
أُذهْوب	/ʔuðhūb/	/ðhb/	2.62	/ʔuCCūC/	2.85	CvC.CVC
تَبلْيد	/tablīd/	/bld/	3.03	/taCCīC/	2.47	CvC.CVC
مَفْكور	/mafkūr/	/fkr/	3.03	/maCCūC/	3.97	CvC.CVC

**Pseudowords R-P+**	Phonology	Root	Root Frequency	Pattern	Pattern Frequency	Structure
نُحاز	/nuħāz/	/nħz/	pseudoroot	/CuCāl/	3.38	Cv.CVC
دَليت	/dalīt/	/dlt/	-0.37	/CaCīC/	4.46	Cv.CVC
باسِك	/bāsik/	/bsk/	-0.34	/CāCiC/	4.57	CV.CvC
رَفاش	/rafāʃ/	/rfʃ/	-0.37	/CaCāC/	4.09	Cv.CVC
دَرود	/darūd/	/drd/	-0.23	/CaCūC/	3.05	Cv.CVC
قَمدَة	/qamdat/	/qmd/	pseudoroot	/CaCCat/	2.68	CvC.CvC
لَمدود	/lamdūd/	/lmd/	-0.48	/CaCCūC/	2.30	CvC.CVC
تَشْداف	/taʃdāf/	/ʃdf/	-0.70	/taCCāC/	2.02	CvC.CVC
تَجْريز	/taʒrīz/	/ʒrz/	-0.64	/taCCīC/	2.47	CvC.CVC
مَدْبوس	/madbūs/	/dbs/	0.11	/maCCūC/	3.97	CvC.CVC

**Pseudowords R-P-**	Phonology	Root	Root Frequency	Pattern	Pattern Frequency	Structure
غيفَة	/ɣīfat/	/ɣf/	-0.89	pseudopattern		CV.CvC
فاخَة	/fāxat/	/fxt/	-0.12	pseudopattern		CV.CvC
ساميج	/sāmīʒ/	/smʒ/	-0.14	pseudopattern		CV.CVC
لوماز	/lūmāz/	/lmz/	-0.16	pseudopattern		CV.CVC
غودَن	/ɣūdan/	/ɣdn/	-0.89	pseudopattern		CV.CvC
سَيْدول	/sajdūl/	/sdl/	-0.16	pseudopattern		CvC.CvC
بَكّيم	/bakkīm/	/bkm/	-0.21	pseudopattern		CvC.CVC
سَلْنَد	/salnad/	/slnd/	-0.18	pseudopattern		CvC.CvC
جَنْدور	/ʒandūr/	/ʒndr/	-0.16	pseudopattern		CvC.CVC
خِرْبَش	/Xirbaʃ/	/Xrbʃ/	-0.18	/CiCCaC/	-0.80	CvC.CvC

**Pseudowords R+P-**	Phonology	Root	Root Frequency	Pattern	Pattern Frequency	Structure
بِحور	/biħūr/	/bħr/	2.83	pseudopattern		Cv.CVC
خوشَب	/Xūʃab/	/Xʃb/	1.84	pseudopattern		CV.CvC
شوكيل	/ʃūkīl/	/ʃkl/	3.28	pseudopattern		CV.CVC
بوعاد	/būʕād/	/bʕd/	3.50	pseudopattern		CV.CVC
هيرَب	/hīrab/	/hrb/	2.23	pseudopattern		CV.CvC
نَيْزول	/najzūl/	/nzl/	2.57	pseudopattern		CvC.CVC
فَتْحين	/fatħīn/	/ftħ/	2.81	pseudopattern		CvC.CVC
أَرْكيز	/ʔarkīz/	/rkz/	3.12	pseudopattern		CvC.CVC
تُحْكيم	/taħkīm/	/ħkm/	3.23	pseudopattern		CvC.CVC
أَسْموع	/ʔasmūʕ/	/smʕ/	2.26	pseudopattern		CvC.CVC

3.2. Written word comprehension

List 1<br>Clothes		List 2<br>Fruits and vegetables		List 3<br>Animals	
**Word**	**Meaning**	**Word**	**Meaning**	**Word**	**Meaning**
حِذاء	Shoe	حَليب	Milk	نَهْر	River
أَلّعاب	Toys	جَبَل	Mountain	أَسَد	Lion
مِعْطَف	Coat	خَسّ	Lettuce	شَمْس	Sun
ثوم	Garlic	مُفْتاح	Key	هِرّ	Cat
فُسْتان	Dress	خَرَز	Pearls	ديك	Rooster
مِظَلّة	Umbrella	تُفّاح	Apple	صوف	Wool
غُيوم	Clouds	جَزَر	Carrot	مَزْرَعة	Farm
بَنْطَلون	Pant	وَرَقَة	Sheet	كَلْب	Dog
فُسْتُق	Groundnut	شَجَرَة	Tree	ذَنَب	Tail
قُبَّعَة	Hat	لَيْمون	Orange	بَقَرَة	Cow
ساعَة	Watch	مَوْزة	Banana	قَلْب	Heart
تَنّورَة	Skirt	وَرْدة	Rose	شَبَكَة	Net
صابون	Soap	خِيار	Cucumber	حَياة	Life
مِحْفَظَة	Wallet	كوب	Cup	نَخْلَة	Palm
ثَوْب	Gown	عِنَب	Grape	سَمَكَة	Fish

3.3. Morphosemantic induction

List	Morphology Type	Stimulus	Phonology	Response	Phonology	Root	Root Frequency	Root Frequency	Stimulus Pattern	Pattern Frequency	Response Pattern	Response Pattern Frequency
1	derivational	شَرَكَ	/ʃaraka/	شَراك	/ʃarrāk/	/ʃrk/	High	3.48	/CaCaCa/	2.98	/CaCCāC/	3.42
1	derivational	كَبَرَ	/kabara/	كَبّار	/kabbār/	/kbr/	High	3.30	/CaCaCa/	2.98	/CaCCāC/	3.42
1	derivational	حَرَبَ	/ħaraba/	حَرّاب	/ħarrāb/	/ħrb/	High	3.11	/CaCaCa/	2.98	/CaCCāC/	3.42
1	derivational	لَمَنَ	/lamana/	لَمّان	/lammān/	/lmn/	High	2.74	/CaCaCa/	2.98	/CaCCāC/	3.42
1	derivational	بَحَتَ	/baħata/	بَحّاتَ	/baħħāt/	/bħt/	Low	0.97	/CaCaCa/	2.98	/CaCCāC/	3.42
1	derivational	حَكَرَ	/ħakara/	حَكّار	/ħakkār/	/ħkr/	Low	0.52	/CaCaCa/	2.98	/CaCCāC/	3.42
1	derivational	مَلَخَ	/malaXa/	مَلّاخ	/mallāX/	/mlX/	Low	0.01	/CaCaCa/	2.98	/CaCCāC/	3.42
1	derivational	فَلَبَ	/falaba/	فَلّاب	/fallāb/	/flb/	Low	pseudoroot	/CaCaCa/	2.98	/CaCCāC/	3.42
2	derivational	عَدِدَ	/ʕadida/	مَعدَد	/maʕdad/	/ʕdd/	High	3.62	/CaCiCa/	3.82	/maCCaC/	3.80
2	derivational	جَنِبَ	/ʒaniba/	مَجنَب	/maʒnab/	/ʒnb/	High	3.41	/CaCiCa/	3.82	/maCCaC/	3.80
2	derivational	بَشِرَ	/baʃira/	مَبشَر	/mabʃar/	/bʃar/	High	2.94	/CaCiCa/	3.82	/maCCaC/	3.80
2	derivational	بَرِكَ	/barika/	مَبرَك	/mabrak/	/brk/	High	2.92	/CaCiCa/	3.82	/maCCaC/	3.80
2	derivational	دَجِلَ	/daʒila/	مَدجَل	/madʒal/	/dʒl/	Low	0.56	/CaCiCa/	3.82	/maCCaC/	3.80
2	derivational	نَفِلَ	/nafila/	مَنفَل	/manfal/	/nfl/	Low	0.29	/CaCiCa/	3.82	/maCCaC/	3.80
2	derivational	دَعِكَ	/daʕika/	مَدعَك	/madʕak/	/dʕk/	Low	-1.52	/CaCiCa/	3.82	/maCCaC/	3.80
2	derivational	سَدِنَ	/sadina/	مَسدَن	/masdan/	/sdn/	Low	-1.52	/CaCiCa/	3.82	/maCCaC/	3.80
3	derivational	شُرْق	/ʃurq/	شَريق	/ʃarīq/	/ʃrq/	High	3.11	/CuCC/	3.19	/CaCīC/	2.89
3	derivational	طُبْع	/ṭubʕ/	طَبيع	/ṭabīʕ/	/ṭbʕ/	High	2.93	/CuCC/	3.19	/CaCīC/	2.89
3	derivational	جُبْل	/ʒubl/	جَبيل	/ʒabīl/	/ʒbl/	High	2.31	/CuCC/	3.19	/CaCīC/	2.89
3	derivational	نُهْر	/nuhr/	نَهير	/nahīr/	/nhr/	High	2.25	/CuCC/	3.19	/CaCīC/	2.89
3	derivational	سُكْف	/sukf/	سَكيف	/sakīf/	/skf/	Low	0.75	/CuCC/	3.19	/CaCīC/	2.89
3	derivational	قُنب	/qunb/	قَنيب	/qanīb/	/qnb/	Low	0.15	/CuCC/	3.19	/CaCīC/	2.89
3	derivational	كُحْت	/kuħt/	كَحيت	/kaħīt/	/kħt/	Low	-1.52	/CuCC/	3.19	/CaCīC/	2.89
3	derivational	شُدْخ	/ʃudX/	شَديخ	/ʃadīX/	/ʃdX/	Low	-1.52	/CuCC/	3.19	/CaCīC/	2.89

4	inflectional	هو سَبَلَ	/sabala/	هي سَبَلَت	/sabalat/	/sbl/	High	2.36				
4	inflectional	هو زَمَلَ	/zamala/	هي زَمَلَت	/zamalat/	/zml/	High	2.12				
4	inflectional	هو كَدَرَ	/kadara/	هي كَدَرَت	/kadarat/	/kdr/	High	1.73				
4	inflectional	هو لَدَنَ	/ladana/	هي لَدَنَت	/ladanat/	/ldn/	High	1.40				
4	inflectional	هو دَبَجَ	/dabaʒa/	هي دَبَجَت	/dabaʒat/	/dbʒ/	Low	0.70				
4	inflectional	هو زَبَدَ	/zabada/	هي زَبَدَت	/zabadat/	/zbd/	Low	0.69				
4	inflectional	هو عَبَكَ	/ʕabaka/	هي عَبَكَت	/ʕabakat/	/ʕbk/	Low	-1.52				
4	inflectional	هو رَخَنَ	/raXana/	هي رَخَنَت	/raXanat/	/rXn/	Low	-1.52				
5	inflectional	هو كَمّال	/kammāl/	هي كَمّالة	/kammālat/	/kml/	High	3.00				
5	inflectional	هو حَصيل	/ħaṣīl/	هي حَصيلَة	/ħaṣīlat/	/ħṣl/	High	2.93				
5	inflectional	هو وسّاع	/wassāʕ/	هي وَسّاعة	/wassāʕat/	/wsʕ/	High	2.83				
5	inflectional	هو قالِم	/qālem/	هي قالِمة	/qālimat/	/qlm/	High	2.81				
5	inflectional	هو رَزيح	/razīħ/	هي رَزيحَة	/razīħat/	/rzħ/	Low	0.26				
5	inflectional	هو دَميل	/damīl/	هي دَميلة	/damīlat/	/dml/	Low	-0.70				
5	inflectional	هو فادِم	/fādem/	هي فادِمة	/fādimat/	/fdm/	Low	-1.52				
5	inflectional	هو تالِع	/tāleʕ/	هي تالِعة	/tāliʕat/	/tlʕ/	Low	-1.52				
6	inflectional	هُوَ يَقدُم	/jaqdumu/	هُم يَقدُمون	/jaqdumūn/	/qdm/	High	3.46				
6	inflectional	هُوَ يَخلَف	/jaXlufuf/	هُم يَخلَفون	/jaxlufūn/	/xlf/	High	3.21				
6	inflectional	هُوَ يَهنِكُ	/jahniku/	هُم يَهنِكون	/jahnikūn/	/hnk/	High	3.16				
6	inflectional	هُوَ يَفرَقُ	/jafraqu/	هُم يَفرَقون	/jafraqūn/	/frq/	High	3.03				
6	inflectional	هُوَ يَبنِفُ	/jabnifu/	هُم يَبنِفون	/jabnifūn/	/bnf/	Low	0.66				
6	inflectional	هُوَ يَرهُمُ	/jarhumu/	هُم يَرهُمون	/jarhumūn/	/rhm/	Low	-1.52				
6	inflectional	هُوَ يَملَجُ	/jamlaʒu/	هُم يَملَجون	/jamlaʒūn/	/mlʒ/	Low	-1.52				
6	inflectional	هُوَ يَعسِبُ	/jaʕsibu/	هُم يَعسِبون	/jaʕsibūn/	/ʕsb/	Low	-1.52				

3.4. Morphophonological induction

List	Stimulus	Phonology	Response	Phonology	Root	Root Frequency	Root Frequency	Stimulus Pattern	Pattern Frequency	Response Pattern	Response Pattern Frequency
1	خُرْج	/Xurʒ/	خَروج	/Xarūʒ/	/Xrʒ/	High	3.38	/CuCC/	3.19	/CaCūC/	3.05
1	سُبْق	/subq/	سَبوق	/sabūq/	/sbq/	High	3.16	/CuCC/	3.19	/CaCūC/	3.05
1	وُصْل	/wuṣl/	وَصول	/waṣūl/	/wṣl/	High	3.13	/CuCC/	3.19	/CaCūC/	3.05
1	دُفْع	/dufʕ/	دَفوع	/dafūʕ/	/dfʕ/	High	3.09	/CuCC/	3.19	/CaCūC/	3.05
1	ثُرْد	/θurd/	ثَرود	/θarūd/	/θrd/	Low	-1.52	/CuCC/	3.19	/CaCūC/	3.05
1	شُرْغ	/ʃurɣ/	شَروغ	/ʃarūɣ/	/ʃrɣ/	Low	pseudoroot	/CuCC/	3.19	/CaCūC/	3.05
1	خُسْب	/Xusb/	خَسوب	/Xasūb/	/Xsb/	Low	pseudoroot	/CuCC/	3.19	/CaCūC/	3.05
1	سُمْغ	/sumɣ/	سَموغ	/samūɣ/	/smɣ/	Low	pseudoroot	/CuCC/	3.19	/CaCūC/	3.05
2	جَدَدَ	/ʒadada/	جادِد	/ʒāded/	/ʒdd/	High	3.50	/CaCaCa/	2.98	/CāCiC/	3.09
2	عَنَدَ	/ʕanada/	عانِد	/ʕāned/	/ʕnd/	High	2.89	/CaCaCa/	2.98	/CāCiC/	3.09
2	طَرَفَ	/ṭarafa/	طارِف	/ṭāref/	/ṭrf/	High	2.82	/CaCaCa/	2.98	/CāCiC/	3.09
2	دَرَبَ	/daraba/	دارِب	/dāreb/	/drb/	High	2.64	/CaCaCa/	2.98	/CāCiC/	3.09
2	بَتَكَ	/bataka/	باتِك	/bātek/	/btk/	Low	-0.44	/CaCaCa/	2.98	/CāCiC/	3.09
2	رَحَسَ	/raħasa/	راحِس	/rāħes/	/rħs/	Low	pseudoroot	/CaCaCa/	2.98	/CāCiC/	3.09
2	رَسَدَ	/rasada/	راسِد	/rāsed/	/rsd/	Low	pseudoroot	/CaCaCa/	2.98	/CāCiC/	3.09
2	عَرَزَ	/ʕaraza/	عارِز	/ʕārez/	/ʕrz/	Low	pseudoroot	/CaCaCa/	2.98	/CāCiC/	3.09
3	جِلَس	/ʒilas/	أَجْلَس	/ʔaʒlas/	/ʒls/	High	3.35	/CiCaC/	2.61	/ʔaCCaC/	4.04
3	وِقَت	/wiqat/	أَوْقَت	/ʔawqat/	/wqt/	High	3.13	/CiCaC/	2.61	/ʔaCCaC/	4.04
3	شِخَص	/ʃiXas/	أَشْخَص	/ʔaʃXas/	/ʃXs/	High	3.04	/CiCaC/	2.61	/ʔaCCaC/	4.04
3	رِسَم	/risam/	أَرْسَم	/ʔarsam/	/rsm/	High	2.97	/CiCaC/	2.61	/ʔaCCaC/	4.04
3	تِمَك	/timak/	أَتْمَك	/ʔatmak/	/tmk/	Low	-1.52	/CiCaC/	2.61	/ʔaCCaC/	4.04
3	مِكَل	/mikal/	أَمْكَل	/ʔamkal/	/mkl/	Low	pseudoroot	/CiCaC/	2.61	/ʔaCCaC/	4.04
3	جِنَف	/ʒinaf/	أَجْنَف	/ʔaʒnaf/	/ʒnf/	Low	pseudoroot	/CiCaC/	2.61	/ʔaCCaC/	4.04
3	مِخَت	/miXat/	أَمْخَت	/ʔamXat/	/mXt/	Low	pseudoroot	/CiCaC/	2.61	/ʔaCCaC/	4.04

4	بَلِغَ	/baliɣa/	بيلَغ	/bīlaɣ/	/blɣ/	High	3.00	/CaCiCa/	3.82	/CīCaC/	pseudopattern
4	شَعِرَ	/ʃaʕira/	شيعَر	/ʃīʕar/	/ʃʕr/	High	3.02	/CaCiCa/	3.82	/CīCaC/	pseudopattern
4	سَبِعَ	/sabiʕa/	سيبَع	/sībaʕ/	/sbʕ/	High	2.99	/CaCiCa/	3.82	/CīCaC/	pseudopattern
4	سَفِرَ	/safira/	سيفَر	/sīfar/	/sfr/	High	2.89	/CaCiCa/	3.82	/CīCaC/	pseudopattern
4	رَبِقَ	/rabiqa/	ريبَق	/rībaq/	/rbq/	Low	-0.07	/CaCiCa/	3.82	/CīCaC/	pseudopattern
4	سَحِمَ	/saħima/	سيحَم	/sīħam/	/sħm/	Low	-0.14	/CaCiCa/	3.82	/CīCaC/	pseudopattern
4	رَزِدَ	/razida/	ريزَد	/rīzad/	/rzd/	Low	pseudoroot	/CaCiCa/	3.82	/CīCaC/	pseudopattern
4	تَمِدَ	/tamida/	تيمَد	/tīmad/	/tmd/	Low	pseudoroot	/CaCiCa/	3.82	/CīCaC/	pseudopattern
5	دِخْل	/diXl/	دوخال	/dūXāl/	/dXl/	High	3.38	/CiCC/	4.67	/CūCāC/	pseudopattern
5	نِتْج	/nitʒ/	نوتاج	/nūtāʒ/	/ntʒ/	High	3.00	/CiCC/	4.67	/CūCāC/	pseudopattern
5	ظِهْر	/ðihr/	ظوهار	/ðūhār/	/ðhr/	High	3.00	/CiCC/	4.67	/CūCāC/	pseudopattern
5	صِرْف	/ṣirf/	صوراف	/ṣūrāf/	/ṣrf/	High	2.97	/CiCC/	4.67	/CūCāC/	pseudopattern
5	مِسْر	/misr/	موسار	/mūsār/	/msr/	Low	-0.41	/CiCC/	4.67	/CūCāC/	pseudopattern
5	شِنْف	/ʃinf/	شوناف	/ʃūnāf/	/ʃnf/	Low	-1.00	/CiCC/	4.67	/CūCāC/	pseudopattern
5	عِكْش	/ʕikʃ/	عوكاش	/ʕūkāʃ/	/ʕkʃ/	Low	-1.15	/CiCC/	4.67	/CūCāC/	pseudopattern
5	بِلْك	/bilk/	بولاك	/būlāk/	/blk/	Low	pseudoroot	/CiCC/	4.67	/CūCāC/	pseudopattern
6	سَلْمُ	/salmu/	أَسْلوم	/ʔaslūm/	/slm/	High	3.65	/CaCCu/	2.34	/ʔaCCūC/	pseudopattern
6	صَبْحُ	/ṣabħu/	أَصْبوح	/ʔaṣbūħ/	/ṣbħ/	High	2.95	/CaCCu/	2.34	/ʔaCCūC/	pseudopattern
6	قَرْبُ	/qarbu/	أَقْروب	/ʔaqrūb/	/qrb/	High	2.93	/CaCCu/	2.34	/ʔaCCūC/	pseudopattern
6	مَكْنُ	/maknu/	أَمْكون	/ʔamkūn./	/mkn/	High	2.88	/CaCCu/	2.34	/ʔaCCūC/	pseudopattern
6	لَحْبُ	/laħbu/	أَلْحوب	/ʔalħūb/	/lħb/	Low	-1.15	/CaCCu/	2.34	/ʔaCCūC/	pseudopattern
6	حَفْنُ	/ħafnu/	أَحْفون	/ʔaħfūn/	/ħfn/	Low	-1.15	/CaCCu/	2.34	/ʔaCCūC/	pseudopattern
6	رَبْلُ	/rablu/	أَرْبول	/ʔarbūl/	/rbl/	Low	-1.52	/CaCCu/	2.34	/ʔaCCūC/	pseudopattern
6	سَتْلُ	/satlu/	أَسْتول	/ʔastūl/	/stl/	Low	-1.52	/CaCCu/	2.34	/ʔaCCūC/	pseudopattern

3.5. Letter naming

ـا - تـ - جـ - ذ - شـ - قـ - خ - سـ - ط - غ – ي – د - عـ - لـ - نـ - ه – ظ - حـ - ثـ - غ - كـ - صـ - ر -
بـ - ض - فـ - و - مـ - يـ - ز

3.6. Phoneme discrimination

Training*:* sat/ʃat - θīl/tīl - ʃif/Xif - dāf/dāf - ʒūl/ ʒūl - naħ/naħ

Experimental:

Different pairs: /sān-zān / - /ʃāħ-ʒāħ/ - /xīb-ɣīb/ - /tāf-dāf/ - /ṭāħ-ḍāħ/ - /sīr-ṣīr/ - /ðūr-ð.ūr/ - /zīf-ð.īf/ - /dūr-ḍūr/ - /tūr-ṭūr/ - /kīm-qīm/ - /tɛl-tal/ - /ṣāl-ṣal/ - /rūf-ruf/ - /Xīl-Xil/ - /θūl-sūl/ - /sāb-ðāb/ - /sīm-ʃīm/ - /fīr-θīr/ - /rīb-līb/ - /mās-lās/ - /nād-lād/ - /ħūl-hūl/ - /kūʃ-tūʃ/ - /ʃur-ʒur/ - /tab-dab/ -/ duɣ-ḍuɣ/ - /riʕ-liʕ/ - /kal-qal/ - /san-zan/ - /θul-sul/

Identical pairs: /tām-tām/ - /sāX-sāX/ - /ṭāf-ṭāf/ - /Xīl-Xīl/ - /ʃān-ʃān/ - /būʕ-būʕ/ - /ṣāf/-ṣāf/ - /dūm-dūm/ -/bur-bur/ - /kīr-kīr/ - /ðūf-ðūf/ - /zīt-zīt/ - /lɛb-lɛb/ - /ras-ras/ - /ħīl-ħīl/ - /dus-dus/ - /ʒāħ-ʒāħ/ - /fās-fās/ - /ʕīb-ʕīb/ - /θaf-θaf/ - /nāʔ-nāʔ/ - /ɣir-ɣir/ - /qub-qub/ - /saf-saf/ - /hid-hid/ - /ḍir-ḍir/- /dan-dan/ - /lad-lad/ - /mik-mik/ - /ṣul-ṣul/

3.7. Phoneme deletion

CVC syllables with long vowels
Training: /ʃār/ –/līm/ – /mūl/ -/kāb/
Experimental: /būʕ/ - /nāX/ - /ʒīn/ – /ħāb/ – /sūr/ – /Xīf/ – /rūb/ - /ʕīn/ - /ḍāħ/ - /jūX/

CVC syllables with short vowels
Training: /ḍaʕ/ - /ʒum/ - /ʕad/ - /tib/
Experimental: /laħ/ – /sut/ - /ṭaf/ - /ʃur/ – /kah/ – /mil/ – /hib/ – /dir/ – /war/ - /ɣuf/

CVCC syllables
Training: /madr/, /lesm/, /fand/, /siʒl/
Experimental: /falb/ - /neʕb/ - /meṣf/ – /tahr/ - /ʒofr/ - /boʃṭ/ - /bajl/ - /maɣl/ – /tikr/ - /fuḍl/

3.8. Rapid naming

Non-repeated condition
Training : /banadūra/ - /dajne/ - /ʔarnab/ - /masṭara/ - /neʒme/ - /warde/ - /manʃafe/ - /kebbēje/ - /ʃawke/ - /tannūra/ - /ʒaras/ - /baṭṭiX/ - /dʒēʒe/ - /mʔaṣ/ - /ṣabūne/ - /ʃanta/ - /ṣūra/ - /mebrējē/ - /seʒʒēde/ - /ʔeṣbaʕ/ - /ʔannīne/ - /lajmūn/ - /baʔra/ - /ɣajme/

Experimental : /ʃamʕa/ - /ṭijjāra/ - /ʔīd/ - /sijjāra/ - /ṭābe/- /taXt/ - /teffēħa/ - /Xjāra/ - /kalsēt/ - /baṭaṭa/ - /ṭawle/ - /ʃams/ - /dūlēb/ - /bēt/ - /meħħāje/ - /bsajne/ - /malʕaʔa/ - /senn/ - /berrad/ - /ʕēn/ - /samke/ - /ʃaʒra/ - /temm/ - /bēb/

Repeated condition
Training : /banadūra/ - /tannūra/ - /lajmūn/
Experimental : /dūlēb/ - /sijjāra/ - /Xjāra/
